# Identifying the Prognosis Factors in Death after Liver Transplantation via Adaptive LASSO in Iran

**DOI:** 10.1155/2016/7620157

**Published:** 2016-08-25

**Authors:** Hadi Raeisi Shahraki, Saeedeh Pourahmad, Seyyed Mohammad Taghi Ayatollahi

**Affiliations:** Department of Biostatistics, School of Medicine, Shiraz University of Medical Sciences, Shiraz, Iran

## Abstract

Despite the widespread use of liver transplantation as a routine therapy in liver diseases, the effective factors on its outcomes are still controversial. This study attempted to identify the most effective factors on death after liver transplantation. For this purpose, modified least absolute shrinkage and selection operator (LASSO), called Adaptive LASSO, was utilized. One of the best advantages of this method is considering high number of factors. Therefore, in a historical cohort study from 2008 to 2013, the clinical findings of 680 patients undergoing liver transplant surgery were considered. Ridge and Adaptive LASSO regression methods were then implemented to identify the most effective factors on death. To compare the performance of these two models, receiver operating characteristic (ROC) curve was used. According to the results, 12 factors in Ridge regression and 9 ones in Adaptive LASSO regression were significant. The area under the ROC curve (AUC) of Adaptive LASSO was equal to 89% (95% CI: 86%–91%), which was significantly greater than Ridge regression (64%, 95% CI: 61%–68%) (*p* < 0.001). As a conclusion, the significant factors and the performance criteria revealed the superiority of Adaptive LASSO method as a penalized model versus traditional regression model in the present study.

## 1. Introduction

Liver transplantation is recognized as a well-established therapy for patients with acute liver failure [1–3]. Despite the fact that it has become widespread and recently the number of liver transplants throughout the world has exceeded 15000 cases in a year, the clinical effective risk factors on liver transplantation outcome are still controversial [4].

Logistic regression is the most common method for assessing the effects of various factors on the binary outcome [5]. Usually, in order to avoid modeling bias, at the initial stage of modeling, a high number of variables are candidates [6]. But logistic regression may encounter with multicollinearity problem (strong correlation between two or more than two independent variables in regression models) in modeling the relation among a high number of variables [7, 8]. In these settings, Ridge regression is a traditional remedial method which can control multicollinearity by imposing a slight bias in the estimation of coefficients. Penalized regressions have recently developed models in facing high dimensional data. Imposing a penalty on the coefficients in penalized methods, besides controlling the multicollinearity, represents a sparse and interpretable model [9]. For instance, least absolute shrinkage and selection operator (LASSO), as one of the most famous penalized models, is applicable regardless of the number of variables and sample size [10].

In some of the previous researches in liver disease, penalized methods were applied and superiority of them versus conventional statistical methods was confirmed by some authors [11, 12]. On the other hand, although the risk of death and their associated effective factors after liver transplantation was investigated in some studies, due to the limitations of conventional statistical methods, a few potential factors were considered. Recently, penalized regression had been widely used in medical sciences for modeling and identifying the most important factors. However, artificial neural networks (ANNs) are frequently used as the nonparametric substituted modeling methods in the issue of large sample size and a high number of variables [2].

Therefore, the aim of this study was to identify the prognosis factors in death after liver transplantation among 35 factors, using logistic Ridge regression and logistic Adaptive LASSO which is a modified version of LASSO with weighted penalties [13].

## 2. Methods

Clinical findings of 680 patients undergoing liver transplant surgery were collected in a historical cohort study from 2008 to 2013 at Nemazee Hospital Organ Transplantation Center, Shiraz, southern Iran. Exclusion criteria were transplantation more than once, less than one day of survival, or any kinds of rejection of transplantation. Independent variables (risk factors) included recipient sex, age, weight, diagnosis disease, comorbidity disease, end-stage liver disease (MELD), or pediatric end-stage liver disease (PELD) score, child class, type of transplantation, previous abdominal surgery, renal failure before and after transplantation, diabetes after transplantation, vascular complication after transplantation, primary nonfunction (PNF), posttransplant lymphoproliferative disorder (PTLD), cytomegalovirus (CMV), lung complication after transplantation, bile duct complication after transplantation, exploration after transplantation, child score, waiting list time (day), creatinine, INR, total bilirubin, cold ischemia time (hour), total bleeding (mL), pack cell (bag), duration of operation (hour), duration of hospital stay (day), donor sex, age, and status while the binary dependent variable (response, event) was death due to liver transplantation during the period of five years after the surgery (yes: 1 and no: 0).

### 2.1. Statistical Analyses

In order to identify the most effective factors on death after liver transplantation, we implemented Ridge and Adaptive LASSO regression. In this article, inverse LASSO coefficients were used for each variable as their weight in Adaptive LASSO. The performance of these two models in classification of high and low risk patients was then compared by calculating the areas under the curve (AUC) in receiver operating characteristic (ROC) curve. Statistical analysis was performed using SPSS 20.0, MedCalc 14.0,* parcor *and* ridge *packages in R 3.1.3 software.

## 3. Results

The patients' age ranged from 2 to 74 years with a mean (SD) of 33.6 (18.27) including 430 (63.2%) males and 250 (36.8%) females. The results revealed that, among 680 patients, only 78 (11.47%) died due to complications from liver transplantation and the others (88.53%) were alive.  Table 1 shows the qualitative characteristics of recipients and quantitative information is displayed in Table 2.

**Figure 1:**
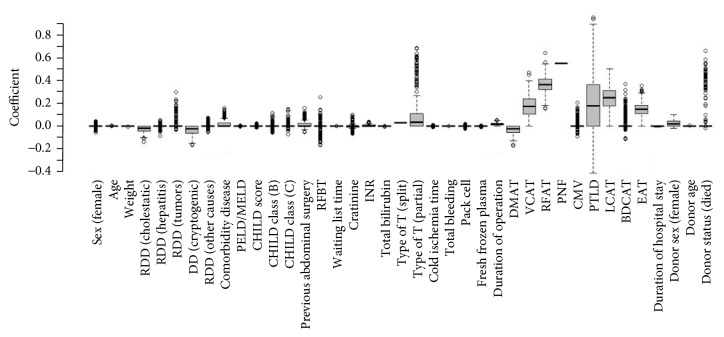
The coefficients of Adaptive LASSO in 500 times bootstrap method. RDD: recipient diagnosis disease, MELD: model for end-stage liver disease, PELD: pediatric end-stage liver disease, RFBT: renal failure before transplantation, DMAT: diabetes mellitus after transplantation, VCAT: vascular complication after transplantation, RFAT: renal failure after transplantation, PNF: primary nonfunction, CMV: cytomegalovirus, PTLD: posttransplant lymphoproliferative disorder, LCAT: lung complication after transplantation, BDCAT: bile duct complication after transplantation, EAT: exploration after transplantation.

Considering the final outcome as dependent variable and all 35 mentioned factors in Tables 1 and 2 as independent variable, Ridge regression and Adaptive LASSO regression were fitted and standard error of coefficients was obtained using 500 times bootstrap method. Among these 35 factors, 12 factors in Ridge regression were significant and 9 ones in Adaptive LASSO regression had nonzero coefficient.  Table 3 represents the results of fitting these two models and Figure 1 displays the coefficients of Adaptive LASSO regression for each factor in bootstrap method. In order to compare the performances of these two models, the values of *p*_*i*_ (risk of death due to complications from liver transplantation) were calculated using the reported coefficients in Table 3 for each model and then patients with high and low risk of death were classified using optimal cut-off point in ROC curve.

**Figure 2:**
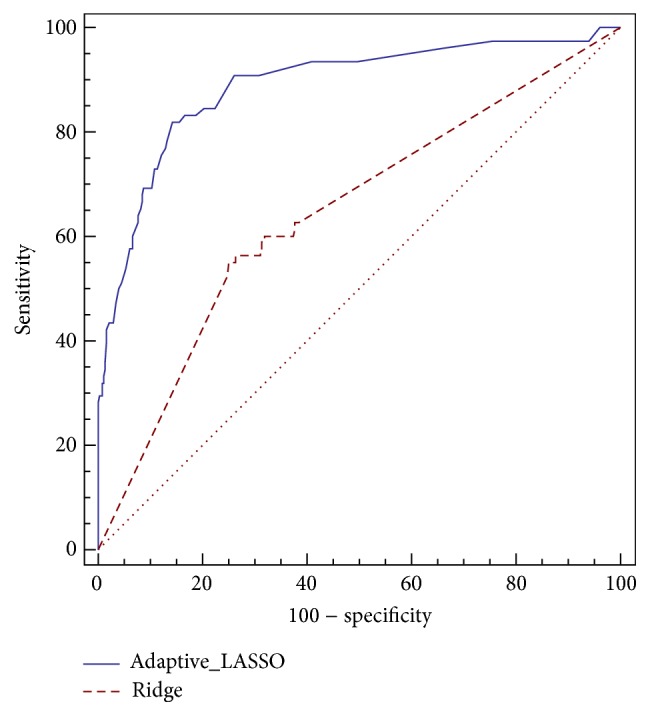
The area under the ROC curve for Ridge and Adaptive LASSO models.

Adaptive LASSO revealed that the risk of death after transplantation in patients with PNF was 1.75-fold compared to the others. Also, renal failure after transplantation with relative risk of 1.42, lung complication after transplantation with relative risk of 1.26, and PTLD with relative risk of 1.23 were introduced as the most important risk factors which can increase the risk of death (Table 3). The AUC of our proposed penalized model was equal to 89% (95% CI: 86%–91%), which was significantly greater than Ridge regression (68%, 95% CI: 64%–71%) (*p* < 0.001). Also, sensitivity and specificity for Adaptive LASSO were 82% and 86%, respectively (Table 4).  Figure 2 compares the ROC curves of two utilized models.

## 4. Discussion

Clinical findings of 680 patients undergoing liver transplant surgery by two considered methods revealed that PNF, PTLD, renal failure, and lung complication after transplantation were the most important prognosis factors in death after liver transplantation with different coefficients or relative risks (Table 3).

Although Ridge regression is known as an effective remedial method for controlling multicollinearity, large values of standard errors and unrealistic coefficients in the second and third columns of Table 3 confirm the presence of multicollinearity in this model. On the other hand, sufficiently small values of standard errors of coefficients in Adaptive LASSO demonstrate controlling the multicollinearity. By omitting the redundant factors, Adaptive LASSO could estimate the risk of death much better than traditional logistic Ridge regression (Table 4). The current study showed that Adaptive LASSO, as a penalized regression, was better than the Ridge regression model in predicting the risk of death in patients after liver transplantation based on the area under the ROC curve (Figure 2 and Table 4).

In our study, PNF was the most important prognosis factor in death after transplantation; this result is in agreement with most of the previous studies about survival after transplantation or risk factors of death after transplantation [14]. It may be due to direct association between PNF and complexity of the surgical procedure that can increase the risk of death [15].

Recently, several studies about the role of renal failure, as an important prognosis factor in death, in hepatitis and kidney disease have been performed [16, 17]. Also, the association of renal failure with increase of morbidity and mortality after heart or liver transplantation in some studies was approved; this is consistent with our findings [18, 19]. In addition, lung and vascular complication after transplantation in our proposed models were represented as two effective factors in death which are considered as two common and main causes of death after liver transplantation [20–22]. In a study about complications and mortality after liver transplantation, both univariate and multivariate analyses revealed a significant association between vascular complication and mortality of recipients [23]. As another result of the current study, like the other studies, we found that PTLD can increase the risk of death [24, 25]. And finally in contrast to our study, factors like the recipient's age, comorbidity disease, previous abdominal surgery, and MELD were reported as death risk factors after transplantation [2, 14, 26]. As a limitation of this study, we can refer to the low number of death (11%) due to the nature of the data.

## 5. Conclusion

To the best of our knowledge, our study was one of the first researches in Iran which considered simultaneous effect of 35 potential factors on the risk of death after transplantation by two powerful statistical methods. Adaptive LASSO regression demonstrates superiority of penalized models versus traditional regression models in facing a high number of variables.

## Figures and Tables

**Table 1:** Descriptive statistics of qualitative variables of 680 patients with liver transplantation utilized in modeling process.

Characteristic	Number (%)
Recipient sex	
Male	430 (63.2)
Female	250 (36.8)
Recipient diagnosis disease	
Metabolic	102 (15)
Cholestatic	140 (20.6)
Hepatitis	267 (39.3)
Tumors	9 (1.3)
Cryptogenic	46 (6.8)
Other causes	116 (17.1)
Comorbidity disease	
No	573 (84.3)
Yes	107 (15.7)
MELD/PELD score	
<20	308 (45.3)
≥20	372 (54.7)
Child class	
A	81 (11.9)
B	305 (44.9)
C	294 (43.2)
Type of transplantation	
Whole	573 (84.3)
Split	36 (5.3)
Partial	71 (10.4)
Previous abdominal surgery	
No	590 (86.8)
Yes	90 (13.2)
Renal failure before transplantation	
No	646 (95)
Yes	34 (5)
Diabetes after transplantation	
No	528 (77.6)
Yes	152 (22.4)
Vascular complication after transplantation	
No	646 (95)
Yes	34 (5)
Renal failure after transplantation	
No	624 (91.8)
Yes	56 (8.2)
PNF	
No	669 (98.4)
Yes	11 (1.6)
PTLD	
No	672 (98.8)
Yes	8 (1.2)
CMV	
No	651 (95.7)
Yes	29 (4.3)
Lung complication after transplantation	
No	655 (96.3)
Yes	25 (3.7)
Bile duct complication after transplantation	
No	664 (97.6)
Yes	16 (2.4)
Exploration after transplantation	
No	567 (83.4)
Yes	113 (16.6)
Donor sex	
Male	452 (66.5)
Female	228 (33.5)
Donor status	
Living	70 (10.3)
Died	610 (89.7)

**Table 2:** Descriptive statistics of quantitative variables of 680 patients with liver transplantation utilized in modeling process.

Characteristic	Mean (SD)
Recipient age (year)	33.6 (18.24)
Weight (kg)	58.67 (23.30)
Child score	9.08 (2.18)
Waiting list time (day)	167.63 (224.87)
Creatinine (mg/dL)	0.89 (0.59)
INR	1.98 (1.22)
Total bilirubin (mg/dL)	8.14 (10.36)
Cold ischemia time (hour)	6.76 (3.46)
Total bleeding (mL)	16.99 (1633)
Pack cell (bag)	2.40 (2.83)
Fresh frozen plasma (bag)	3.19 (4.01)
Duration of operation (hour)	6.03 (1.28)
Duration of hospital stay (day)	12.84 (7.73)
Donor age (year)	31.2 (15.25)

**Table 3:** Coefficients of nonzero factors in Ridge and Adaptive LASSO logistic regression.

Characteristic	Method				
	Ridge regression		Adaptive LASSO regression		
	Coefficient	SE	Coefficient	SE	RR
PNF	8.51	1.98	0.56	0.10	1.75
Renal failure after transplantation	13.59	1.86	0.35	0.07	1.42
Lung complication after transplantation	6.42	1.85	0.23	0.10	1.26
PTLD	3.70	1.74	0.21	0.22	1.23
Vascular complication after transplantation	6.49	1.80	0.17	0.10	1.19
Exploration after transplantation	9.93	1.92	0.14	0.06	1.15
Type of transplantation					
Whole	Baseline	—	Baseline	—	—
Split	4.06	1.89	0.00	0.06	1.00
Partial	2.96	1.26	0.03	0.15	1.03
Duration of operation (hour)	6.31	1.97	0.02	0.01	1.02
Donor sex					
Male	Baseline	—	Baseline	—	—
Female	4.25	2.03	0.02	0.02	1.02
Donor age	4.45	1.93	0.00	0.01	1.00
Diabetes after transplantation	−4.02	1.89	0.00	0.03	1.00

**Table 4:** Sensitivity, specificity, and AUC of Ridge regression and Adaptive LASSO methods in modeling effective factors on death of 680 patients after liver transplantation.

Method	AUC (95% CI)	Sensitivity (95% CI)	Specificity (95% CI)
Ridge	67.7 (64–71.2)	84.6 (74.7–91.8)	46.7 (42.6–50.8)
Adaptive LASSO	89 (86.4–91.2)	82.1 (71.7–89.8)	85.5 (82.5–88.3)
